# Genomic DNA Methylation-Derived Algorithm Enables Accurate Detection of Malignant Prostate Tissues

**DOI:** 10.3389/fonc.2018.00100

**Published:** 2018-04-23

**Authors:** Erfan Aref-Eshghi, Laila C. Schenkel, Peter Ainsworth, Hanxin Lin, David I. Rodenhiser, Jean-Claude Cutz, Bekim Sadikovic

**Affiliations:** ^1^Department of Pathology and Laboratory Medicine, Western University, London, ON, Canada; ^2^Molecular Genetics Laboratory, Molecular Diagnostics Division, London Health Sciences, London, ON, Canada; ^3^Department of Pediatrics, Western University and Children’s Health Research Institute, London, ON, Canada; ^4^Department of Biochemistry, Western University and Children’s Health Research Institute, London, ON, Canada; ^5^Department of Oncology, Western University and Children’s Health Research Institute, London, ON, Canada; ^6^Department of Pathology and Laboratory Medicine, McMaster University, Hamilton, ON, Canada

**Keywords:** prostate cancer, DNA methylation, protein interaction, transcription factor binding, copy number variation, differentially methylated regions, machine learning, LASSO

## Abstract

**Introduction:**

The current methodology involving diagnosis of prostate cancer (PCa) relies on the pathology examination of prostate needle biopsies, a method with high false negative rates partly due to temporospatial, molecular, and morphological heterogeneity of prostate adenocarcinoma. It is postulated that molecular markers have a potential to assign diagnosis to a considerable portion of undetected prostate tumors. This study examines the genome-wide DNA methylation changes in PCa in search of genomic markers for the development of a diagnostic algorithm for PCa screening.

**Methods:**

Archival PCa and normal tissues were assessed using genomic DNA methylation arrays. Differentially methylated sites and regions (DMRs) were used for functional assessment, gene-set enrichment and protein interaction analyses, and examination of transcription factor-binding patterns. Raw signal intensity data were used for identification of recurrent copy number variations (CNVs). Non-redundant fully differentiating cytosine-phosphate-guanine sites (CpGs), which did not overlap CNV segments, were used in an L1 regularized logistic regression model (LASSO) to train a classification algorithm. Validation of this algorithm was performed using a large external cohort of benign and tumor prostate arrays.

**Results:**

Approximately 6,000 probes and 600 genomic regions showed significant DNA methylation changes, primarily involving hypermethylation. Gene-set enrichment and protein interaction analyses found an overrepresentation of genes related to cell communications, neurogenesis, and proliferation. Motif enrichment analysis demonstrated enrichment of tumor suppressor-binding sites nearby DMRs. Several of these regions were also found to contain copy number amplifications. Using four non-redundant fully differentiating CpGs, we trained a classification model with 100% accuracy in discriminating tumors from benign samples. Validation of this algorithm using an external cohort of 234 tumors and 92 benign samples yielded 96% sensitivity and 98% specificity. The model was found to be highly sensitive to detect metastatic lesions in bone, lymph node, and soft tissue, while being specific enough to differentiate the benign hyperplasia of prostate from tumor.

**Conclusion:**

A considerable component of PCa DNA methylation profile represent driver events potentially established/maintained by disruption of tumor suppressor activity. As few as four CpGs from this profile can be used for screening of PCa.

## Introduction

Prostate cancer (PCa) is the most common malignancy in men, representing the third greatest cause of mortality in the general population after lung and colorectal cancer (1). Current screening protocols for PCa rely on digital rectal examination along with the evaluation of serum levels of prostate-specific antigen. If a tumor is suspected, trans-rectal, ultrasound-guided, prostate needle biopsies are conducted, followed by a histopathology examination. Major limitations of this approach include a relatively high false negative rate and the requirement for annual follow-ups and repeat sampling, an invasive approach with significant burden on patient health care and lifestyle (2). As such, a significant effort has been made to identify more accurate diagnostic and screening biomarkers for PCa, though to date this effort has been met with limited success in clinical application (3).

Among the markers that have gained interest in disease screening is DNA methylation, an epigenetic mechanism that includes modification of the fifth base of cytosine at cytosine-phosphate-guanine (CpG) dinucleotide in the DNA by addition or removal of a methyl group. DNA methylation plays an integral role in the regulation of gene expression and genomic stability, disruption of which can lead to neoplastic transformation, carcinogenesis, and cancer progression (4). The relative stability of the DNA molecule together with the genome-wide distribution of DNA methylation marks has made it an attractive target for disease biomarker discovery (5). DNA methylation has proven to possess a great potential in the prediction of many human traits and conditions, including age, postpartum depression, childhood stress exposure, tumor class, response to therapy, and cancer progression/metastasis (5).

Epigenetic changes have been previously demonstrated in multiple cancers including PCa (6), with epigenomic signatures of PCa being proposed to be used as both diagnostic and prognostic markers (3, 7). PCa-associated DNA methylation changes have also been found in the urine and serum of patients, raising hopes for non-invasive methods of PCa screening (3). These methylation changes have been found in various genes involved in hormonal response, cell-cycle regulation, cell invasion, and DNA damage repair (6). In particular, hypermethylation in the promoters of tumor suppressor genes *APC, RARβ*, and *GSTP1*, has been proposed to be used as a diagnostic marker for PCa (8). However, these assays have not generated optimal performances, partly because they target selected candidate genes instead of markers discovered in a genome-wide search. The number of studies attempting to produce a diagnostic methylation algorithm for PCa using a genome-wide approach is limited. Furthermore, few studies have conducted a comprehensive genome-wide DNA methylation analysis to reveal the role of DNA methylation in PCa (3).

In the present study, we have conducted a genome-wide DNA methylation analysis to describe the DNA methylation changes in PCa. We have investigated potential mechanisms by which differentially methylated genes may be involved in the development of PCa, by examining pathway enrichment and protein interactions. In addition, we have addressed mechanisms that regulate the establishment of differentially methylated regions (DMRs) in PCa by investigating the transcription factor (TF)-binding patterns nearby the identified segments. Also, we report the copy number variations (CNVs) recurrently happening in PCa and evaluate their associations with the methylation levels of the overlapping DMRs. Finally, using the most differentiating CpGs, we have generated a classification model to distinguish the tumors from the adjacent benign tissues, and have validated this algorithm using a large independent cohort of benign and tumor prostate samples.

## Materials and Methods

### Specimen Collection

Archival formalin-fixed paraffin-embedded tissues were collected from the Department of Pathology at McMaster University, ON, Canada. The slides were reviewed by a pathologist to identify representative tumor tissue blocks. Enrichment of tumor cells was performed by tissue macro-dissection of predetermined areas, as outlined by Haemotoxylin and Eosin staining. Seven-micron thick sections in isolated areas of interest with at least 50% tumor cellularity were dissected to be used as tumor samples. Normal prostate samples were obtained from the adjacent tissues demonstrating no tumor involvement.

### Methylation Assay and Quality Assessment

Genomic DNA extraction was conducted using the Illumina FFPE DNA recovery kit. Following bisulfite conversion, DNA methylation analysis of the samples was performed using the Illumina Infinium methylation 450 k bead chip array (San Diego, CA, USA), according to the manufacturer’s protocol. Both tumor and normal samples were assayed in one experiment to avoid batch effect. The resulting methylated and unmethylated signal intensity data were imported into R 3.4.2 for analysis. Normalization was performed using Illumina normalization method with background correction using the *minfi* package (9). Probes with detection *p*-value > 0.01, and those known to contain single nucleotide polymorphisms at the CpG interrogation or single nucleotide extension were removed. All of the samples were examined for genome-wide methylation density, and those deviating from a bimodal distribution were excluded. Factor analysis using multiple dimensional scaling did not reveal any unexplained variation or outliers among the samples. The raw and processed genome-wide methylation microarray data has been deposited to gene expression omnibus (GEO) (accession ID: GSE112047).

### Identification of a PCa-Related Methylation Profile

DNA methylation analysis was performed using a modification of our previously published protocol (10–15). Methylation level for each probe was measured as a beta value, calculated from the ratio of the methylated signals vs. the total sum of unmethylated and methylated signals, ranging between 0 (no methylation) and 1 (full methylation). This value was used for biological interpretation and visualization. For statistical analysis, beta values were logit transformed to *M*-values using the following equation: log2 (beta/1 − beta). A linear regression modeling using the *limma* package (16) was used to identify the differentially methylated probes by testing the association of every CpG site with the level of tumor involvement (0–90%). The generated *p*-values were moderated using the *eBayes* function in the *limma* package and were corrected for multiple testing using *Bonferroni* method. Probes with a corrected *p*-value < 0.01 and a methylation difference > 20% were considered significant. The effect size cut-off (20%) was determined following the examination of the volcano plot of the analysis, as conducted in our previous study (17). The identified probes were examined using an unsupervised hierarchical clustering to ensure their ability in separating the tumors from normal samples.

### Gene Enrichment Analysis and Identification of Differential Methylation Interaction Hotspots

To identify the gene ontology (GO) terms overrepresented in the genes harboring differentially methylated probes, a gene-set enrichment analysis was performed using the *missMethyl* package (18), taking into account the number of CpG sites per gene. GO terms with a *Bonferroni* corrected *p*-value <0.01 were considered significant. Only the biological processes were reported. The redundant GO terms were reduced, and following a multiple dimensional scaling, visualized using *REViGO* tool (19). The relationships across the significant GO terms were plotted in a hierarchical order using *GORILLA* tool (20).

We used *EpiMod* algorithm (21) to search for the “interactome hotspots” of differential promoter methylation. In this algorithm, protein expression changes are inferred according to a model of inverse association between the promoter methylation and gene expression. Among the differentially expressed genes in an interactive network, a hotspot [or epigenetic module (EpiMods)] is a sub-network with an exceptionally large average edge-weight density (combined methylation statistics of the neighboring genes) as compared to the rest of the network (21). To assign a statistical significance to the identified hotspots, 1,000 Monte Carlo randomization of the molecular profiles were conducted as suggested by the algorithm. Interactive network hotspots composed of at least ten genes and FDR < 0.01 were reported.

### Identification of DMRs in PCa

To identify genomic regions harboring methylation changes (DMRs), a bump-hunting approach was used by the *bumphunter* package (22). The analysis considered regions with >20% change in the overall methylation between tumor and normal samples with gaps no more than 500 bp among neighboring CpGs. As suggested by the package, 1,000 bootstrapping procedures were performed to compute family-wise error rate (FWER). We selected regions containing a minimum of three consecutive probes and FWER < 0.01. The identified regions were mapped to CpG islands and coding genes. *Gviz* package was used for visualization of the DMRs.

### Identification of Copy Number Changes Using DNA Methylation Array

To estimate the copy number alterations in the prostate tumor from the Infinium methylation array, the raw methylated and unmethylated intensities from every sample were summed, and quantile normalized using the *preprocessCore* package (23). The normalized matrix values were divided by the median values of every probe across the normal samples. The divided ratios were then log10 transformed, smoothed, and segmented using the *DNAcopy* Bioconductor package (24) to identify genomic regions in every sample showing a copy number change. A *p*-value of <0.01 obtained from 10,000 permutations was used to define a change point during segmentation. The segmented regions with a minimum of five probes and an average log ratio > 0.2 or <−0.3 (corresponding to at least one copy amplification/deletion) were selected. Using the *GenomicRanges* package (25), the segments with a similar pattern of copy number change in both normal and tumor samples were excluded. Segments on chromosome six, overlapping HLA genes, were not considered due to normal variations in their copy numbers. From the remaining segments, those occurring in more than one tumor sample were reported.

### TF-Binding Site Enrichment Analysis in DMRs

We investigated the enrichment of TF-binding motifs in both the identified DMRs and their immediate surroundings (±5 kb) using the pipeline recommended by the *RTFBSDB* package (26). First, the entire direct and inferred Homo sapiens TF-binding motifs were downloaded from the *Cis-BP* database (27). Every DMR sequence and its ±5 kb surroundings were scanned for motif enrichment relative to a background genome of 100,000 base pairs. As recommended by the package, the difference in the GC content of the DMRs and the background genome was accounted for by re-sampling of the background genome to avoid a potential bias due to GC content. The motifs with an enrichment ratio ≥1.5 folds and a *Bonferroni* corrected *p*-value < 0.01 were selected for assessment.

### Construction and Validation of a DNA Methylation-Based Diagnostic Model for PCa

The differentially methylated probes were used to build a classification model to differentiate tumor samples from the normal tissues. Only CpG probes located outside the regions with copy number change were utilized for feature selection. First, a receiver operating characteristic curve analysis (ROC) was performed to identify the most differentiating probes. Those probes with an area under the curve (AUC) of 1.00 were retained. Next, pairwise correlations among the remaining probes were measured to identify and exclude the redundant features with *R*-squared >0.90. A least absolute shrinkage and selection operator (LASSO) was used to further narrow down the features and train the model, using the *glmnet* package (28). Following 1,000 permutations, the shrinkage parameter of lambda with lowest misclassification error was selected and incorporated into the final model. The model was set to generate probability scores ranging 0–1, representing the chance of a given sample being a tumor. The default binary classifier’s probability threshold of 0.5 was used as classification cut-off. To assess the performance of the model, publically available methylation data from normal and tumor prostate tissues, benign prostate hyperplasia (BPH), and samples of PCa metastasis in other tissues were downloaded from GEO and blindly supplied to the model. Based on the scores and classifications produced by the algorithm for these samples, we measured accuracy, sensitivity, specificity, and AUC as indices of performance for our classifier. The GEO accession IDs and their related publications of the methylation data files used for validation of our algorithm are mentioned here to give credit to the researchers providing these data. Tumor and adjacent benign array files were downloaded from GSE76938 (29), GSE52955 (30), GSE47915 (31), GSE55479 (32), GSE83917 (32), and GSE38240 (33). The normal prostate array data from radical prostatectomy due to bladder cancer, and those with BPH were obtained from GSE55599 (34). Methylation data of tissues with metastases from prostate were included in the files from GSE38240 (33).

### Ethics Statement

This study has been approved by the Hamilton Integrated Research Ethics Board (#14-700-T). All of the samples and records were de-identified prior to analysis.

## Results

### Study Cohort

The study cohort included 31 samples of archival prostate gland tissue with a confirmed diagnosis of prostate adenocarcinoma, assessed by a licensed pathologist. From 16 of these, adjacent normal tissues were collected to be used as the control group. The tumor sections were dissected ensuring a minimum of 50% cancer involvement using macrodissection. There was no overall difference between the age of the subjects in the control group (55.7 ± 7.3) and the tumor group (55.2 ± 9.9).

### PCa Generates a Hypermethylation Profile

**Figure 1:**
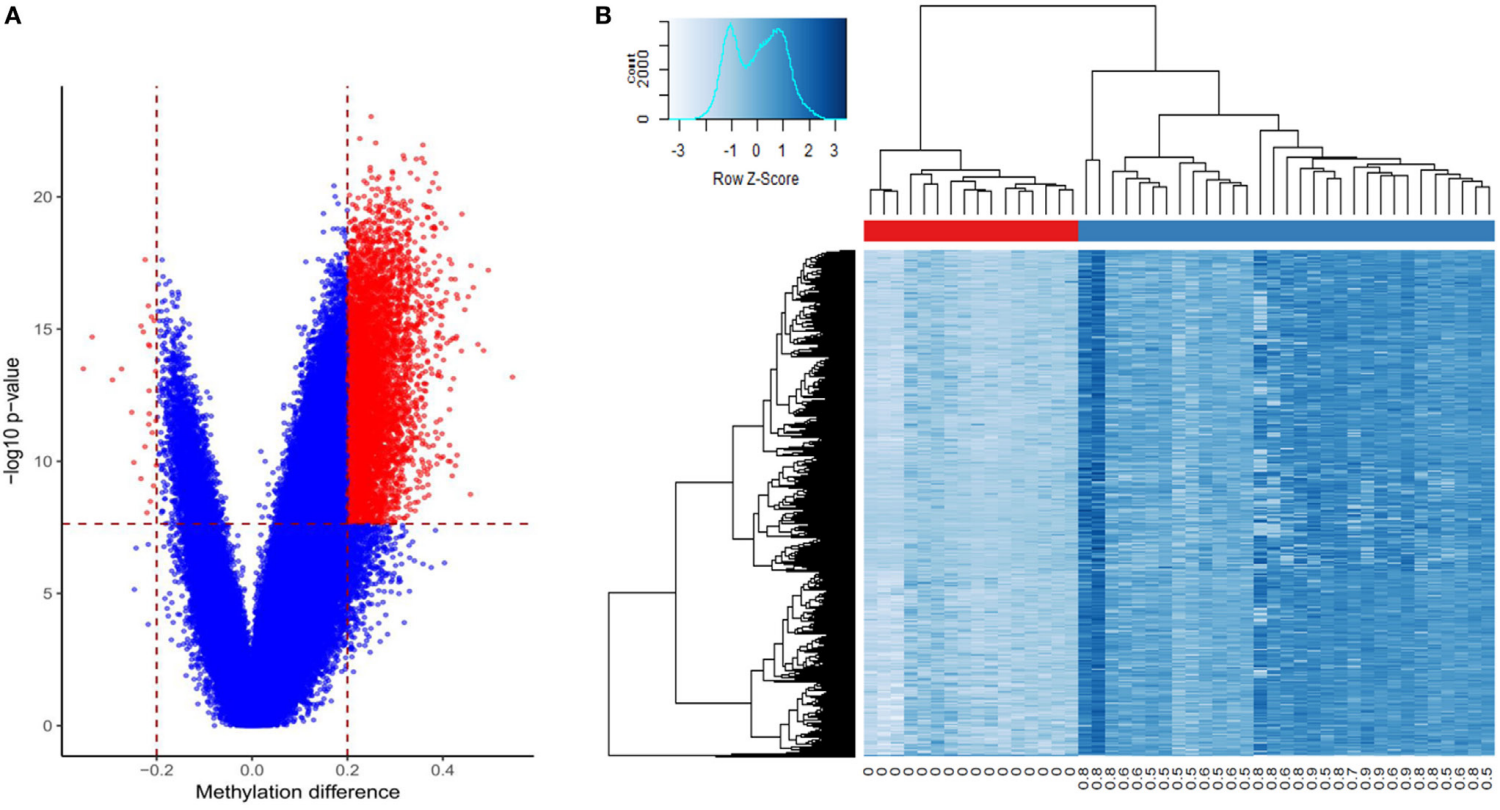
Differentially methylated cytosine-phosphate-guanine (CpG) sites in prostate cancer: **(A)** volcano plot of the comparison between the tumors and benign samples: *X*-axis: methylation difference (mean tumor − mean normal); *Y*-axis: negative logarithmic scale of *p*-value; vertical dashed lines: methylation difference cut-off (0.2); horizontal line: *p*-value cut-off (0.01, Bonferroni-corrected). The significant probes are shown in red; **(B)** Heatmap of the tumors (columns, blue bar), and the adjacent benign tissues (columns, red bar) using 6,167 differentially methylated loci (rows): intensity of blue color corresponds to the methylation levels. Numbers bellow columns: level of tumor cellularity.

From a total of 440,532 CpG probes that passed the quality controls, a total of 6,167 were revealed to have a methylation difference of >20% between the two groups with a multiple testing corrected *p*-value <0.01 (Table S1 in Supplementary Material; Figure 1A). Out of these CpG sites, only 27 (0.4%) showed a hypomethylation change. The vast majority of these probes were annotated to a coding gene (*n* = 4,334, 70.2%). A significant number showed an association with regulatory features, including 5,011 (81.2%) being located in or nearby a CpG island, 1,959 (31.7%) being annotated to DNase Hypersensitivity Sites, and 2,000 (32.3%) located in an enhancer element. Using an unsupervised hierarchical clustering, these probes were shown to be able to completely separate the two groups. The degree of hypermethylation was observed to be correlated with the level of tumor cellularity (Figure 1B). A correlation analysis between the mean methylation levels of the entire profile and tumor cellularity revealed a Pearson correlation coefficient of 0.92 (Figure S1 in Supplementary Material).

### Cell Communication Is the Central Functional Entity of PCa Methylation Profile

**Figure 2:**
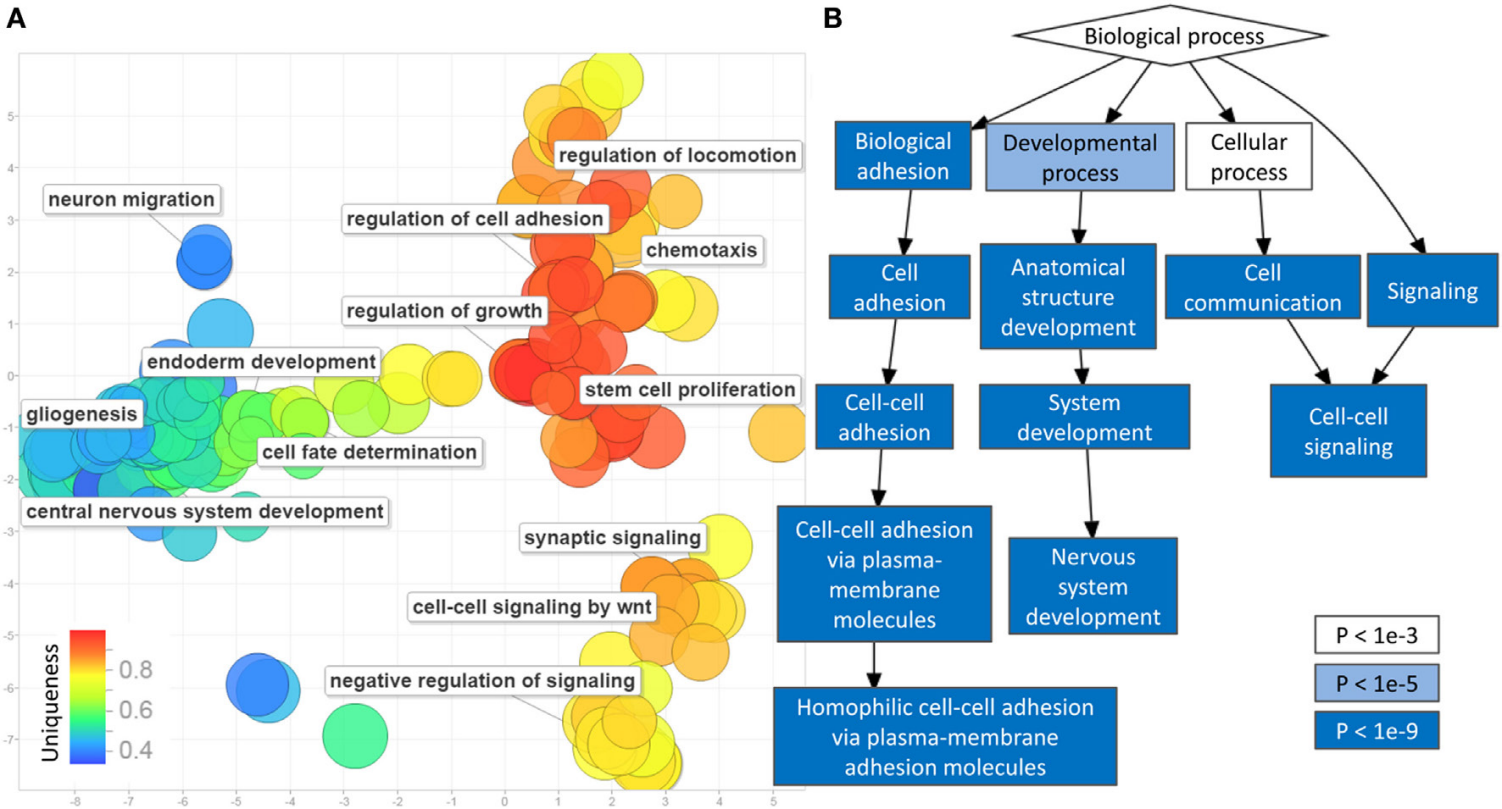
Gene-set enrichment analysis of differentially methylated cytosine-phosphate-guanine sites (CpGs) in prostate cancer: **(A)** multiple dimensional scaling of the gene ontology (GO) terms (circles): GO terms with closely related functionalities are clustered in groups. Circles with smaller distance from each other represent GO terms with similar functionality. The uniqueness of every GO term is shown using a color scale from blue (less unique) to red (more unique). Representative non-redundant GO terms from every cluster are written next to the related circles. Only 350 of the GO terms were selected by REVIGO for reduction and visualization (maximum software limit); **(B)** hierarchical relationship between the significant GO terms: the level of significance (*p*-value) of every functional category is illustrated by a color scale from white to blue.

Gene-set enrichment analysis of the CpG sites with differential methylation identified 466 enriched GO terms (biological processes) with a multiple testing corrected *p*-value < 0.01, considering the number of probes in every gene (Table S2 in Supplementary Material). The most frequent terms in this list include various forms of cell–cell communications, growth, senescence, bone morphogenetic protein signaling, neurogenesis, and differentiation (Figure 2). Protein interaction analysis of the differentially methylated promoters using the *EpiMod* algorithm identified a total of 9 genes as a hotspot for protein–protein interactions containing a minimum of 10 interacting partners and an FDR < 0.01 (Tables S3 and S4 in Supplementary Material; Figure S2 in Supplementary Material). Among these are genes known to be involved in carcinogenesis, including C-X-C motif chemokine ligand 5 (*CXCL5*), estrogen-related receptor gamma (*ESRRG*), and sclerostin domain containing 1. The most active hotspot, however, was found for collagen type-3 alpha-1, located at the center of an interactive network of *COL5A2, COL5A1, COL11A1, COL11A2, PCOLCE*, and *HTRA1*, all being members of extracellular matrix regulation pathways (Table S4 in Supplementary Material; Figure S2 in Supplementary Material).

### Genomic Regions Differentially Methylated (DMRs) in PCa

Using a bump hunting approach, we found 613 genomic coordinates containing a minimum of three consecutive CpGs, an average regional methylation difference >0.2, and a FWER < 0.01 (Table S5 in Supplementary Material). All of these regions were hypermethylated, and the vast majority was mapped to coding genes and CpG Islands. Among the largest and most differentially methylated segments are hypermethylation in the promoters of tumor suppressors (*APC*, 10 probes, 30%; *KLK10*, 4 probes, 28%), genes involved in regulation of cellular senescence (*HIF3A*, 8 probes, 31%), cell communication and adhesion [protocadherin (PCDH) gene cluster (*n* = 88), 3–9 probes, 21–36%], and genes regulating the growth including *PDE4D* (6 probes, 23%) and estrogen receptor-related gamma (*ESRRG*, 6 probes, 24%). In addition, a number of chromatin regulators such as histone deacetylase 9 (*HDAC9*, 6 probes, 26%) and *TET1* (4 probes, 31%), as well as genes coding for histone subunits (*HIST1H*-*1A, 3G, 4D, and 4F*) were found to show increased methylation in their promoters (3–7 probes, 22–27%).

### Copy Number Changes (CNVs) in PCa Correlate With DMR Status

**Figure 3:**
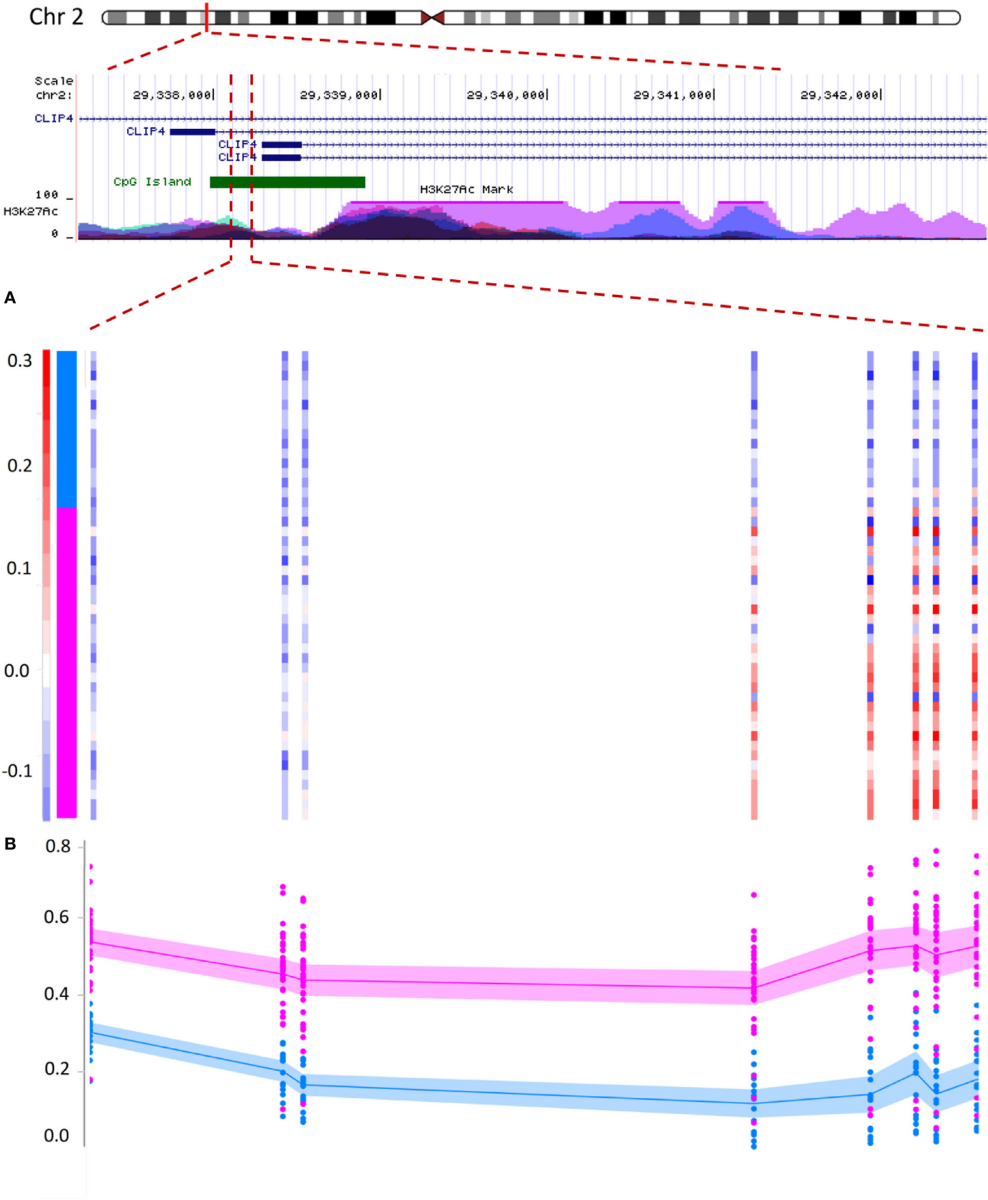
Correlation between copy number variation (CNV) status and DNA methylation in prostate cancer: hypermethylation in the promoter of the *CLIP4* gene partially correlates with its CNV status. The figure illustrates a segment in the promoter of *CLIP4* gene, located in the short arm of chromosome 2. The region is marked with high levels of acetylation of the 27th lysine residue of Histone 3, (H3K27Ac), a maker associated with active promoters. The segment is also recognized as a cytosine-phosphate-guanine (CpG) island (green pane). Panels **(A,B)** represent 181 base-pairs of this segment, harboring a total of 8 CpG probes, which were identified to concurrently show both hypermethylation and CNV amplification. **(A)** The color scale of the vertical bars (each probe) represents the log ratios of the copy numbers. The color scale above 0.2 is shown with red and indicates a minimum of one copy amplification for the region. Color scales of white and light blue represent no CNV change (we defined a CNV loss with a log ratio less than −0.3, which is not observed for this region). Samples are sorted from top to bottom. The top 16 samples represent normal tissues and the lower samples indicate the tumors (vertical pane as an indicator: blue and pink). Within this segment, the right five probes show CNV amplification in tumors, but not in the adjacent benign tissues. **(B)** The methylation status of the same eight probes shows a hypermethylation in tumors (pink) relative to normal adjacent benign tissues (blue). Methylation level of every probe from every sample is presented with a dot, representing a methylation range between 0 and 1 (bottom to top). Lines represent mean, and shadows around the lines indicate 95% confidence intervals of the mean in every group. The region with CNV amplification is significantly hypermethylated; however, this hypermethylation extends beyond the CNV to three probes in the upstream.

Copy number analysis identified a total of 33 segments in the tumor samples containing a minimum of five markers and one copy of loss/gain, observed in more than one tumor (Table S6 in Supplementary Material). Most of these regions were located nearby coding genes. Out of these, two segments, one in chr2: 29338077–29338258 and the other in chr15: 45421860–45422325, annotating to *CLIP4* and *DUOX1*, were most recurrently amplified among the tumors (in seven and eight samples, respectively). Comparison of the CNV and DMR lists revealed that both of these regions also show an increased level of methylation (Table S5 in Supplementary Material; Figure 3). The same incidence was also observed for regions in the promoters of *KRT3* and *PLEC* (each amplified in three tumors), but not among other CNVs. For instance, a segment in the promoter of *PNCK* (amplified in three tumors) did not reveal any hypermethylation change. These results suggested that the hypermethylation pattern seen in some of the DMRs might be associated with their CNV status.

### DMRs in PCa Are Surrounded by Tumor Suppressor-Binding Motifs

**Figure 4:**
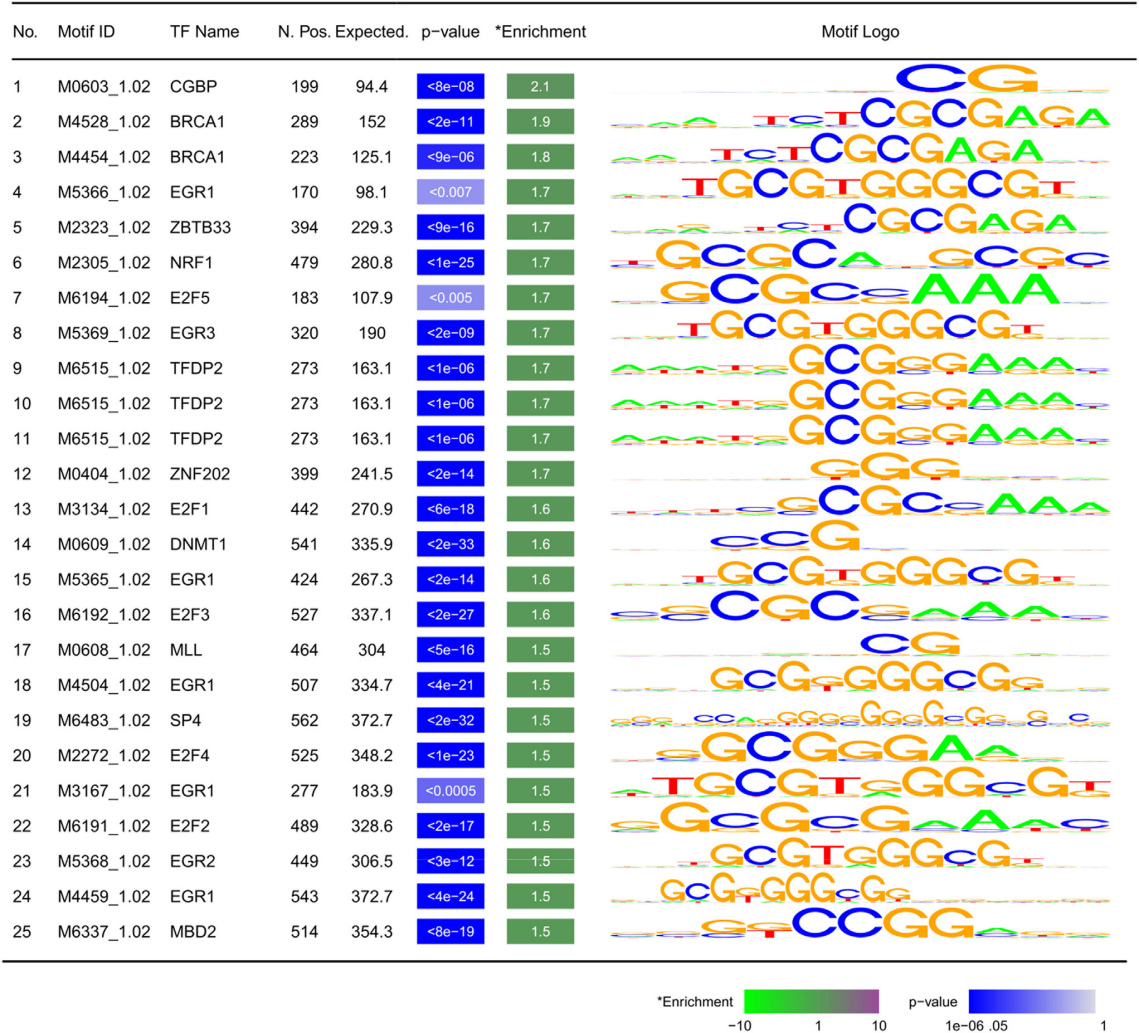
Motifs enriched within ±5 kb of differentially methylated regions: TF, transcription factor; N. Pos.: number of observed motifs; expected: number of expected motifs; enrichment: fold enrichment; *p*-value is corrected for multiple testing using Bonferroni method. Motifs are sorted by fold enrichment.

Analysis of 1,946 Homo-sapiens-specific TF-binding motifs from the Cis-BP database revealed that the DMR sequences rarely bind to any of these TFs. Similarly, a TF-binding site enrichment analysis found an inverse enrichment for any of these motifs among these DMR sequences (enrichment ratios < 1, data not shown). We hypothesized that this might be related to the possibility that DMRs are not a direct target for regulatory elements; rather, they occur as a result of *cis*-regulation by TFs binding to the sites around them. Therefore, we expanded the DMRs by 5 kb upstream and downstream and re-analyzed the data. We found enrichment for 25 motifs specific to 18 TFs (minimum fold change: 1.5, Bonferroni corrected *p*-value < 0.01, Figure 4). Except for few being chromatin regulators (CGBP, MBD2, and DNMT1), all of these TFs were found to have tumor suppressor functionality (BRCA1, E2F1&2&5&7, EGR1-4, FLI1, NRF1, TFDP2, and STAT1), or indirectly mediate the suppression of tumorigenesis and tumor suppressor activity (SP4 and ZBTB33).

### A Four-CpG Classification Model Enables Accurate Diagnosis of PCa

**Figure 5:**
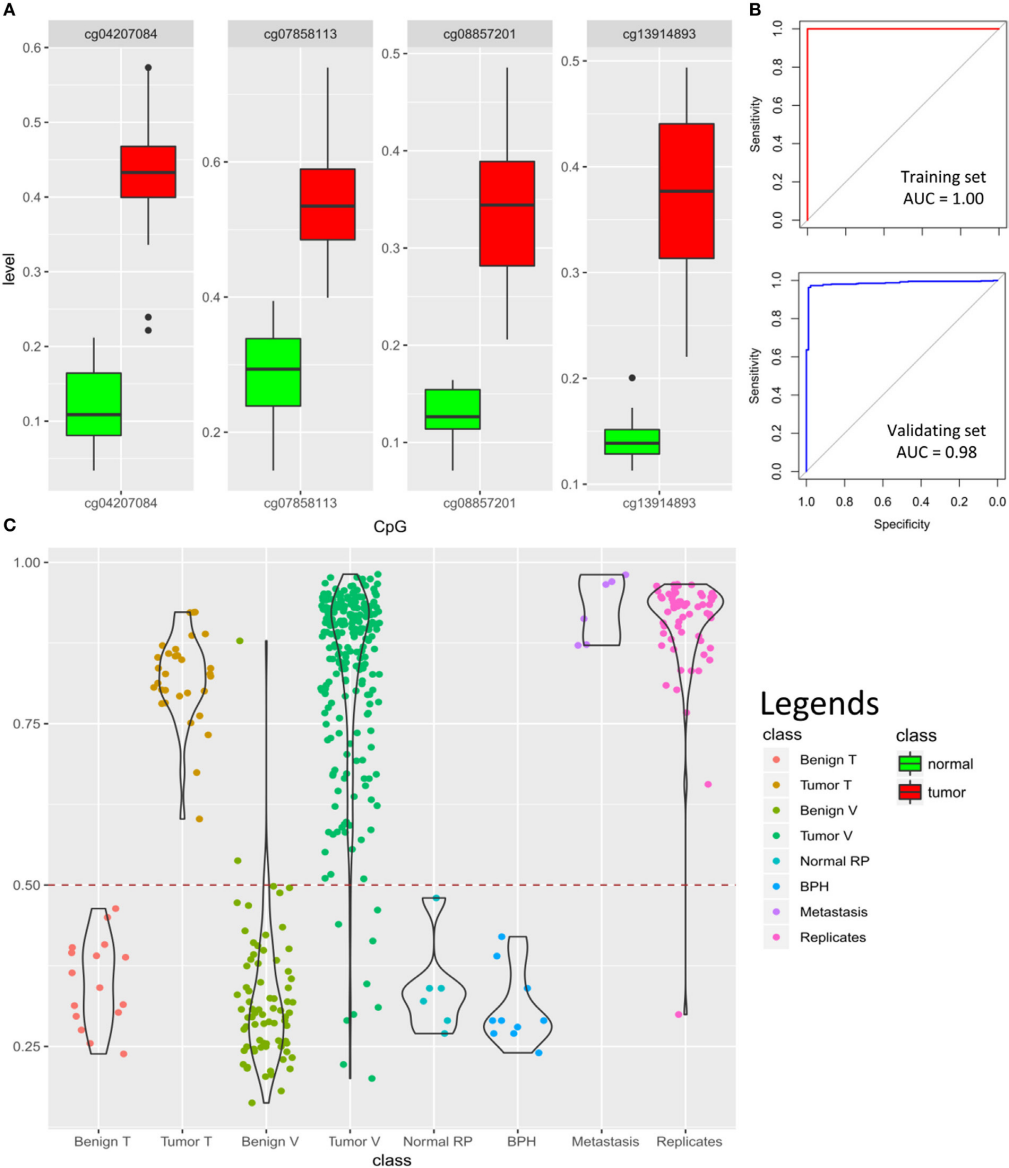
Prediction algorithm for classification of prostate samples: **(A)** four cytosine-phosphate-guanine (CpG) probes selected by LASSO for training the classification model show significant hypermethylation in the tumors as compared to the normal samples. *Y*-axis represents the methylation levels. **(B)** The classification model yields 100% accuracy [area under the curve (AUC) = 1.00] in the training dataset and 97% accuracy (AUC = 0.98) in the validating dataset of 92 benign samples and 234 tumors (model details in Tables S7 and S8 in Supplementary Material); **(C)** classification scores generated by the model for 16 normal samples in the training dataset (Benign T), 31 tumor samples in the training dataset (Tumor T), 76 benign tissue from validating dataset (Benign V), 234 tumors from validating dataset (Tumor V), 6 normal radical prostatectomy from validating dataset (Normal RP), 10 benign prostate hyperplasia (BPH) from validating dataset, 6 prostate cancer metastasis from bone, lymph node, and soft tissue (Metastasis), as well as 61 tumor technical replicates are shown using violin-jitter plots. *Y*-axis represents the tumor probability scores (0–1), stratified by different classes on the *X*-axis. Violin-jitter plots show the density and distributions of the scores in every category. The normal samples mostly receive a score between 0.15 and 0.45, while the majority of the tumors are scored >0.65. The default cut-off of 0.5 (dashed line) is used for classification. Only two of the normal samples out of 92 received a score similar to other tumors, and only 9 misclassifications out of 234 have been made for tumors. Technical replicates have also generated comparable scores to the samples in the original experiment.

To design a classification model with the ability of detecting PCa tumor status from methylation profile, we first performed feature selection from the significant PCa-related probes and then trained a LASSO model with a binary outcome (tumor vs. normal). To avoid a potential bias from the variations in DNA copy numbers, we excluded the probes located in CNV regions. Next, we selected the non-redundant and fully differentiating probes (AUC = 1.00), which resulted in the retention of 46 CpGs. These probes were incorporated into a LASSO model, which following penalization, assigned coefficients equal to 0 to 42 of the probes, dropping them out of the model. Therefore, the final model was trained using four CpGs (Figure 5A) on 16 normal samples and 31 tumors (model details in Table S7 in Supplementary Material). These four CpGs are located in the first intron of *OLFM1*, promoter of *RFX7*, 12th intron of *PTPRN2*, and the promoter of *FLOT1*, respectively (Table S7 in Supplementary Material).

To test the model, we first assessed its performance on the training data. As expected, all of the samples were assigned a correct classification of tumor vs. benign (accuracy, sensitivity, specificity, and AUC of 100%; Figure 5B). Next, to validate our algorithm, we downloaded DNA methylation array data of prostate samples publically available from GEO, and blindly supplied their methylation levels from the four CpGs to our model for classification. Of a total of 234 tumor and 76 benign prostate arrays (adjacent tissue to tumor) that we found in GEO, the algorithm correctly classified 225 as having a tumor profile and 74 as being benign, respectively. Next, we downloaded six normal prostate arrays from radical prostatectomy due to bladder cancer and ten arrays from BPH samples and supplied them to our model, all of which were classified as normal, suggesting that our algorithm can also differentiate BPH from the tumor (Figure 5C). Altogether, this analysis revealed a sensitivity of 0.96 (9 false negatives in 234 tumors), specificity of 0.98 (2 false positives among 74 adjacent benign, 6 BPH, and 10 normal), an overall accuracy of 0.97, and AUC of 0.98 for the validating dataset (Figure 5B). Among other files available from GEO were arrays from bone (*n* = 3), lymph node (*n* = 2), and soft tissue (*n* = 1) with metastases from PCa, all of which were confidently predicted to have a methylation profile similar to PCa. In addition, a total of 61 technical replicates of the prostate tumors were available from datasets GSE83917 and GSE55479. Prediction scores generated for these files remained consistent with their original pairs, including the false negatives, suggesting that our model is not sensitive to technical variation. The prediction scores for all of the above array files are presented in Figure 5C and Tables S8 and S9 in Supplementary Material.

## Discussion

In this report, we have described DNA methylation changes in PCa as compared to the adjacent benign tissue and reported a hypermethylation profile, which is overrepresented in genes and interactive networks that regulate cell–cell signaling, cell communications, growth, and differentiation. Our study has revealed that the DMRs in PCa do not directly overlap regulatory elements; instead, they are surrounded by TF binding motifs specific to tumor suppressors. And finally, we have demonstrated that by using as few as four CpGs one can accurately classify prostate specimens as malignant or benign.

### Insights Into the Involvement of DNA Methylation in PCa

A hypermethylation profile has been reported by previous DNA methylation studies of PCa, which appears to be maintained in all stages of the disease, likely due to selection pressure (29, 35). Previous reports have also found that, in contrast to the hypermethylated CpGs that will gain further methylation during tumor progression, hypomethylation events are less likely to be maintained in the more advanced stages of the disease (33). Consistent with these results, we observed that the level of hypermethylation in PCa directly correlates with the degree of tumor cellularity in the primary prostate specimens (Figure S1 in Supplementary Material). The postulated link between a hypermethylation profile and cancer is a model of translational silencing of tumor suppressors by promoter hypermethylation (6). Indeed, we have observed increased methylation in the promoters of several tumor suppressor genes including *APC* (Figure S3 in Supplementary Material), a well-characterized tumor suppressor gene associated with familial adenomatous polyposis, and *KLK10*, which has been shown to repress proliferation and induce apoptosis in PCa cells (36). Among other DMRs in genes involved in growth and differentiation in our study are *PDE4D*, encoding a signal transduction molecule with cyclic-AMP phosphodiesterase activity that is shown in mice to promote proliferation of PCa (37). As well, we have observed promoter hypermethylation in *ESRRG*, which was also found to act as a hotspot for protein–protein interactions. Downregulation of *ESRRG* has been found in certain types of prostate carcinomas, and it has been shown that its increased expression can repress tumor proliferation regardless of androgen sensitivity (38).

Besides tumor suppressors with a potential role in cell growth and proliferation, analyses of gene-set enrichment and protein–protein interaction hotspots have revealed that methylation changes in PCa encompass a combination of biological processes extending beyond the regulation of growth to neurogenesis and cell–cell communication. The best-known involvement of neurons in cancer is perineural invasions, where tumor invades the neural tissue. Another form of neural involvement in cancer, namely neurogenesis, has been recently reported in prostate tumors as the formation of neural components, axonogenesis, and increased number of neurons (39). Neurogenesis has been found to correlate with both perineural invasions and poor clinical outcomes in PCa (39). Our gene enrichment analysis contains multiple GO terms related to the development of the nervous system, including brain development, axonogenesis, and neurogenesis. In parallel, these analyses provide evidence for the involvement of diverse forms of cell–cell signaling and communications, ranging from regulation of the extracellular matrix to chemotaxis and cellular response to stimuli. Among all of the genes in our results that are known to take part in these processes, the *PCDH* genes are mutually involved in both neurogenesis and cell–cell communications. Our DMRs contain extensive regions that overlap the promoters of close to 90 *PCDH gamma* genes and isoforms. Various members of this cluster have been shown to be present in cell–cell adhesion, neural stroma and synapses (40), and homophilic trans-interactions (41). Overexpression of *PCDHs in vitro* has been demonstrated to suppress Wnt signaling and to inhibit colony formations of cancer cells (42). Limited data suggest an involvement of *PCDH* 8, *11*, and *PC* in PCa; however, no information is available regarding the *PCDH gamma* genes in PCa (43–45). Hypermethylation of various subtypes of *PCDH gamma* promoter is reported in Wilms’ tumor, colorectal carcinoma, uterine leiomyosarcoma, esophageal adenocarcinoma, and Barrett’s esophagus (46).

Overall, the major biological mechanisms that can be inferred from the methylation changes in this study represent the driver events, required for the maintenance, integrity, and survival of the tumor rather than those that may initiate tumorigenesis resulting from the loss of tumor suppressor functions. Similarly, the most recurrent CNV changes in tumors were found as amplifications in the promoters of *DUOX1*, a NADPH oxidase involved in the maintenance of tissue homeostasis (47), as well as *CLIP4*, an intracellular linker protein whose knock-down has been shown to increase cell migration and viability in clear cell renal cell carcinomas (48). These two segments were also found to be hypermethylated, potentially by the expansion of the CG repeats or methylation quantitative trait loci resulting from CNV amplification (4). Therefore, some of the hypermethylation changes we have observed in PCa might be related to the CNV status of the regions. However, the more common phenomenon that is potentially involved in the establishment of the DMRs appears to be a dysregulation following a change in tumor suppressor activity. The interesting finding that almost the entire enriched motifs nearby DMRs are specific to tumor suppressors indicates that DMRs in PCa, which mainly represent cancer driver events, are potentially generated or maintained secondary to a dysregulation in tumor suppressors’ function.

### Clinical Implications of the 4-CpG Classification Model

The current method widely in use for the diagnosis of PCa is an 8–12 core needle biopsy, which is well known to encompass a high false-negative rate (49). A significant number of cancer-free reports of the prostate biopsies are shown to be at risk of having an undiagnosed PCa (49). Approximately 25–50% of these men are diagnosed with PCa in a second biopsy performed within 1 year (50). On the other hand, a positive needle biopsy is not confidently replicable. Serefoglu et al. have shown that a repeat PCa diagnosis can be made only in 67.8% of biopsies from post-operative prostate glands of men undergone radical prostatectomy due to PCa, using the same 12-core mapping as performed pre-operatively (51). The main reason for this discrepancy is the spatiotemporal, molecular, and morphological heterogeneity of the prostate adenocarcinoma that leads to a non-uniform presentation of the tumor by the involved tissue (52). This indicates that a significant portion of negative biopsies can contain molecular changes associated with cancer without completely representing histological features of adenocarcinoma (53, 54), and thus a reliable biological marker would assign a diagnosis to a considerable number of suspicious samples undiagnosed following histopathology examination. Consistent with this, Troyer et al. was among the first to publish a report that DNA methylation markers can detect PCa in histopathologically cancer-negative prostate tissues of men who went on to have subsequently positive biopsies (55). Within a few years of this report, methylation markers in the promoters of *GSTP1, APC*, and *RASSF1* were introduced as a commercial tissue-based assay to identify patients in need of repeat biopsies (56). However, clinical validation studies of this assay have only revealed a sensitivity of 68% and a specificity of 64% (57, 58), questioning the reliability of a candidate gene approach in PCa screening. We have also observed hypermethylation to a variable extent in all these three genes (Tables S1 and S5 in Supplementary Material); however, this is not consistently found across all specimens (Figure S3 in Supplementary Material for APC), thereby resulting in reduced sensitivity for cancer tissue specification. As a consequence, efforts to identify DNA methylation biomarkers shifted toward a genome-wide approach for PCa. Goh et al. (59), following a genome-wide DNA methylation analysis, have proposed a 55-CpG classification model, which has yielded 89.8% sensitivity and 66.7% specificity in the validation step. More recently, Kirby et al. have reported a 3-CpG model for classification of tumor samples from the adjacent benign tissues, which reached a sensitivity of 90% and specificity of 82% as measured in a validation cohort of 49 benign and 213 tumor samples (29).

Here, we have further improved the classification of prostate samples by designing a 4-CpG classification model, which has revealed 100% accuracy in separating tumors from benign tissue in our internal dataset. The validation of this model using heterogeneous cohorts of 326 prostate samples from publically available resources has revealed 96% sensitivity and 98% specificity with an overall accuracy of 97% (AUC = 0.98). To our knowledge, this far supersedes performance of any reported DNA methylation-based algorithm for the diagnosis of PCa. Our model is sensitive enough to detect PCa metastasis in other tissues, including bone, soft tissue, and lymph nodes (Figure 5), while it is still specific enough to discriminate BPH from the tumor. This method can be used to develop a low-cost high-throughput targeted assay for diagnosis of PCa or to complement the pathological examination of cancer-negative needle biopsies. It may also be used for confirmation of metastatic lesions outside of the prostate.

### Future Directions

The current classification algorithm presented in this study is designed and validated using tumor samples and normal adjacent tissues obtained from radical prostatectomy. Before this method is translated into clinical use, further computational training, validation, and clinical trials are required. First, the performance of the model needs to be assessed on samples with diverse ranges of tumor cellularity. In the present study, we did not assess samples with tumor involvement less than 50%, and thus, the accuracy remains unknown with regards to samples with lower levels of tumor cellularity. In a small subset of the tumor samples in the validation dataset, we observed scores close to the ones found in normal tissues, which might be caused by a potentially low level of tumor cellularity in them. Computationally, we can re-train the algorithm to enable the prediction of tumor cellularity as a continuous measure. In addition, the model will have to be tested and potentially re-trained on DNA obtained from needle core biopsies. In order to make this technology more broadly applicable, its accuracy should be demonstrated on limited amounts of DNA obtained from needle biopsy specimens, and address whether the performance is similar across the biopsy specimens and the entire prostate gland. Another important aspect to assess is whether this assay can detect neoplastic transformation in specimens that appear to be normal in an initial pathological examination, but are demonstrated to harbor tumor in subsequent assessments of the same core (i.e., false negatives).

The markers and methodology presented here could be expanded to research applications with the aim of developing novel approaches in the non-invasive screening of PCa using circulating tumor DNA. The study we have described herein reveals the power of high-throughput genomic technologies, in combination with machine learning, to increase our understanding of the biology of cancer and, at the same time, to provide us with novel, precise approaches for cancer screening and diagnosis.

## Ethics Statement

This study has been approved by the Hamilton Integrated Research Ethics Board (#14-700-T). All of the samples and records were de-identified prior to analysis.

## Author Contributions

EA-E: performed statistical and bioinformatics analysis and machine learning, and wrote the manuscript; LS and J-CC: collected the specimens; PA, HL, and DR: commented on the manuscript; BS: oversaw the study protocol.

## Conflict of Interest Statement

The authors declare that the research was conducted in the absence of any commercial or financial relationships that could be construed as a potential conflict of interest.
