# Supplementary material for: Genomic DNA Methylation-Derived Algorithm Enables Accurate Detection of Malignant Prostate Tissues
Source: Front Oncol. 2018 Apr 23;8:100. doi: 10.3389/fonc.2018.00100 (PMC5925605; doi:10.3389/fonc.2018.00100)
Supplement: Supplementary file 1 [file Image_1.PDF]

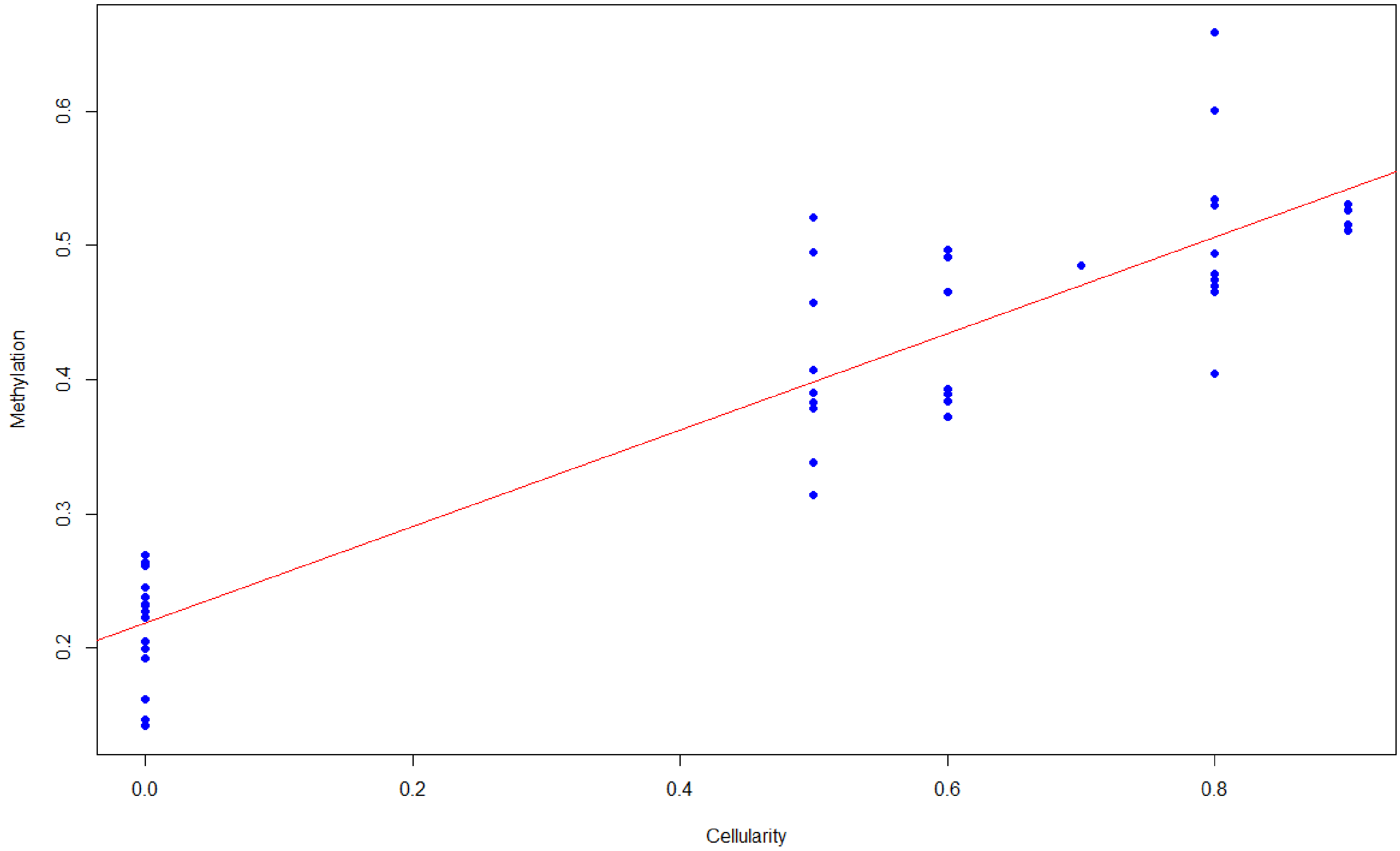

Figure S1- The average methylation levels of the identified probes across the samples (Y-axis) are highly corelated with the level of tumor cellularity (X-axis). Corelation coefficient: 0.92 ($p<0.0001$)

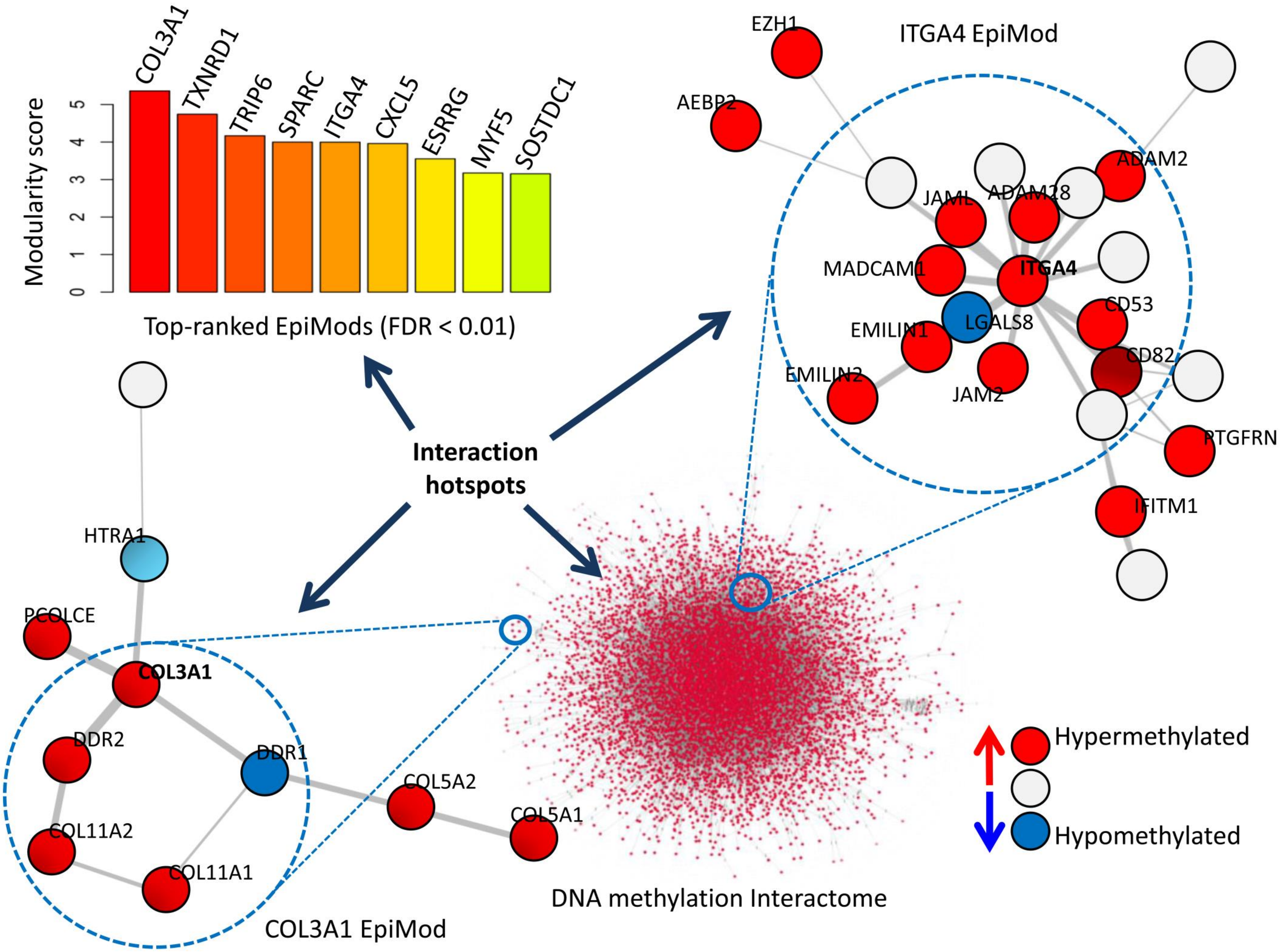

Figure S2- Protein interaction analysis of differentially methylated genes: The DNA methylation protein interactome of the analyzed

genes are composed of a complex network of nodes (proteins encoded by genes) and edges (connecting lines). The edge weight is composed of the combined absolute values of the differential methylation statistics of the probes in the promoters of the two interacting genes. Within this complex network, nine sub-networks with at least ten genes were found to have an average edge weight (modularity) higher than that observed in 1,000 random permutations (FDR<0.01). The modularity scores of these nine hotspots (EpiMods) are presented as a bar plot on the top left. Among these, two EpiMods are visualized (COL3A1 and ITGA4, bottom left and top right). Red and blue nodes represent hyper- and hypo-methylation of the gene promoters, respectively. The white nodes represent genes with no methylation change (details in Tables S3-4).

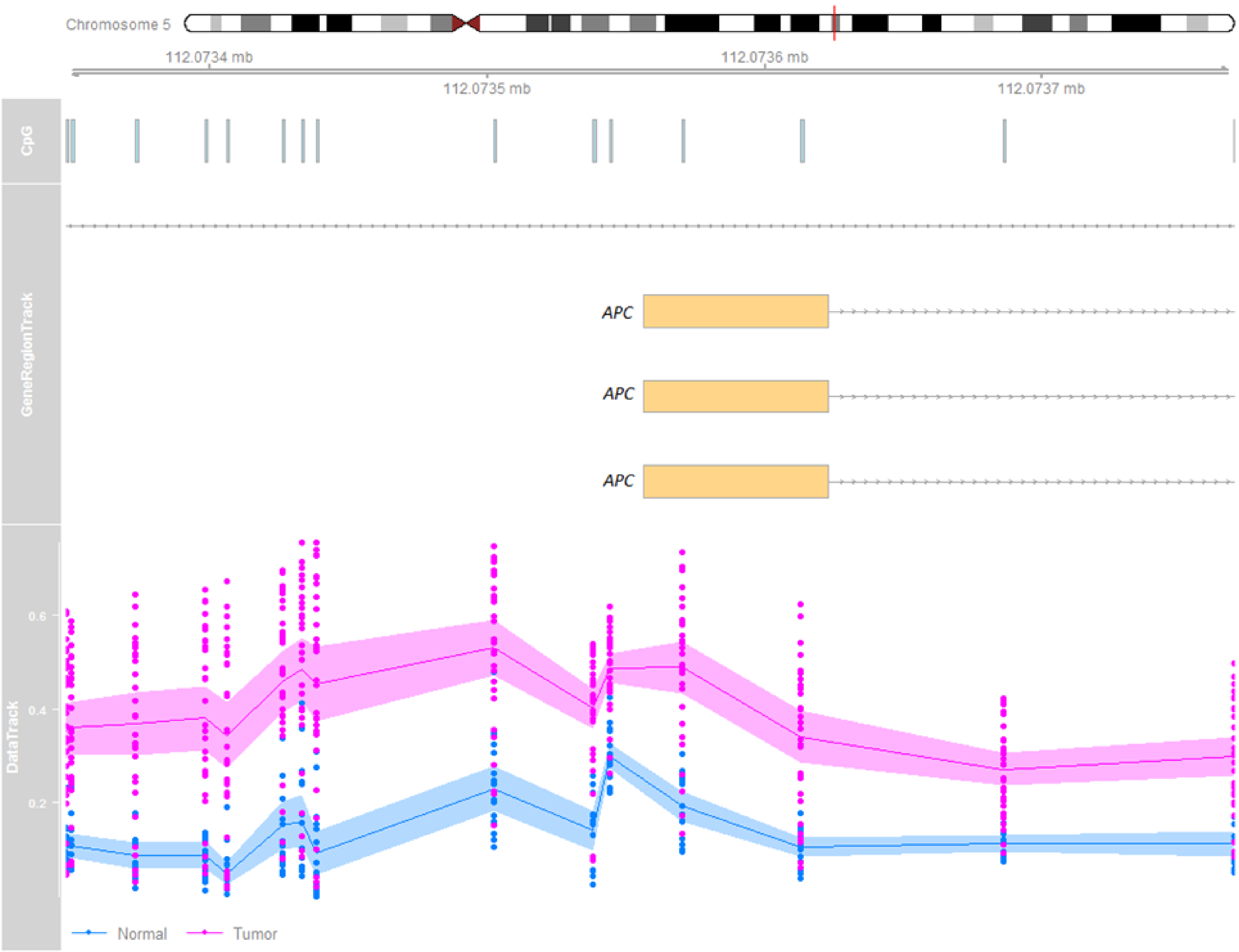

Figure S3- Hypermethylation of APC promoter in 31 prostate cancer FFPE tissues as compared with 16 normal tissues. The figure

illustrates a 422 base-pair region, containing 15 probes with an average methylation difference of 29% between the two groups (family-wise error rate after 1,000 permutations < 0.0001), overlapping 5'UTR, first exon and first intron of APC gene. Track one: Chromosome Ideogram; Track two: CpG probes; Track 3: Gene region; Track Four: Methylation level data; Pink: Tumor; Blue: Normal; Line: average methylation; Shadow: 95% confidence interval; Dots: methylation values from every single sample (0-1).
